# Antibiotic resistance in *Pseudomonas aeruginosa* and adaptation to complex dynamic environments

**DOI:** 10.1099/mgen.0.000370

**Published:** 2020-04-29

**Authors:** Lea M. Sommer, Helle K. Johansen, Søren Molin

**Affiliations:** ^1^​ Department of Clinical Microbiology, Rigshospitalet, 2100 Copenhagen Ø, Denmark; ^2^​ Novo Nordisk Foundation Center for Biosustainability, Technical University of Denmark, 2800 Kongens Lyngby, Denmark; ^3^​ Department of Clinical Medicine, Faculty of Health and Medical Sciences, University of Copenhagen, 2200 Copenhagen N, Denmark

**Keywords:** bacterial pathogens, antibiotic resistance, persistent bacterial infections, *Pseudomonas aeruginosa*, genomics, phenomics

## Abstract

Antibiotic resistance has become a serious threat to human health (WHO *Antibacterial Agents in Clinical Development: an Analysis of the Antibacterial Clinical Development Pipeline, Including Tuberculosis*. Geneva: World Health Organization; 2017), and the ability to predict antibiotic resistance from genome sequencing has become a focal point for the medical community. With this genocentric prediction in mind, we were intrigued about two particular findings for a collection of clinical *Pseudomonas aeruginosa* isolates (Marvig *et al*. *Nature Genetics* 2015;47:57–64; Frimodt-Møller *et al*. *Scientific Reports* 2018;8:12512; Bartell *et al*. *Nature Communications* 2019;10:629): (i) 15 out of 52 genes found to be frequently targeted by adaptive mutations during the initial infection stage of cystic fibrosis airways (‘candidate pathoadaptive genes’) (Marvig *et al*. *Nature Genetics* 2015;47:57–64) were associated with antibiotic resistance (López-Causapé *et al*. *Fronters in Microbiology* 2018;9:685; López-Causapé *et al*. *Antimicrobal Agents and Chemotherapy* 2018;62:e02583-17); (ii) there was a parallel lack of resistance development and linkage to the genetic changes in these antibiotic-resistance-associated genes (Frimodt-Møller *et al*. *Scientific Reports* 2018;8:12512; Bartell *et al*. *Nature Communications* 2019;10:629). In this review, we highlight alternative selective forces that potentially enhance the infection success of *P. aeruginosa* and focus on the linkage to the 15 pathoadaptive antibiotic-resistance-associated genes, thereby showing the problems we may face when using only genomic information to predict and inform about relevant antibiotic treatment.

## Data Summary

The primary data supporting this mini-review has been reported in the following papers: Marvig *et al.* (2015) DOI: 10.1038/ng.3148, Frimodt-Møller *et al*. (2018) DOI: 10.1038/s41598-018-30972-y and Bartell *et al*. (2019) DOI: 10.1038/s41467-019-08504-7.

**Impact Statement**

As more genomes are being sequenced every day (human as well as bacterial), it has become increasingly enticing to focus on what can be predicted from these genomes, e.g. disease predispositions, treatment opportunities and antibiotic resistance to mention a few. However, in spite of being increasingly aware of the complexity of predicting traits from genetic changes, many are still firm believers that we will soon be able to predict the best treatment opportunities, for example, for bacterial infections from the genomic content of said bacteria. This paper critically reviews the opinion that antibiotic resistance can be predicted solely from the genetic changes observed in the bacteria *Pseudomonas aeruginosa *. This is done because of the contradictory findings of three papers focusing either purely on genetics or a combination of genetics and phenotypic traits in a collection of clinical isolates of *P. aeruginosa *. The contradiction is the presence of antibiotic-resistance-associated genetic changes and the lack of antibiotic resistance in the bacterial collection. Thus, we highlight the problems that we may face if we only rely on genetic information to inform about relevant antibiotic treatment.

## Introduction

Antibiotic resistance is defined here by the European Committee on Antimicrobial Susceptibility Testing (EUCAST) determinations of clinical breakpoints. In the clinic, a bacterium is defined as being resistant when the measured minimum inhibitory concentration (MIC) is above the breakpoint. This is in contrast to ‘decreased susceptibility’ or ‘low-level resistance’, which is observed as an increase in MIC compared with previous measurements that is below the EUCAST breakpoint and, therefore, which is generally not reported in the medical clinic.

**Fig. 1.**
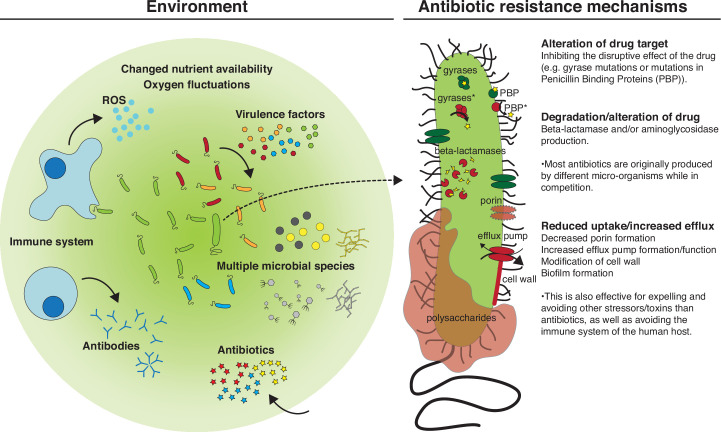
The complex environment of a human host (left) and the main mechanisms applied by *P. aeruginosa* to become antibiotic resistant (right). The bullet points on the right explain how antibiotic-resistance mechanisms also provide survival mechanisms against other stressors and/or toxins found in the environment apart from antibiotics. ROS, Reactive oxygen species. Asterisks indicate mutated versions of the proteins in question.

Antibiotic resistance is either acquired (horizontal gene transfer) or evolved (mutational resistance), and includes three main mechanisms: (i) alteration of antibiotic targets, (ii) degradation or chemical modification of antibiotics, and (iii) reduced uptake or increased efflux across the bacterial cell wall (Fig. 1). These mechanisms are often governed by several genes and often associated with other bacterial traits that are important for survival in the human host.

Here, we will discuss resistance mutations appearing during adaptive evolution of *Pseudomonas aeruginosa* in airway infections of antibiotic-treated cystic fibrosis (CF) patients [1–3]. We will, in particular, focus on 52 genes recently identified as the most frequently mutated genes, which are, therefore, assumed to be important for early adaptation to the CF airways (pathoadaptive genes) [1]. The study provided a comprehensive genomic analysis of the initial 0–10 years of infection by *P. aeruginosa* in 34 young CF patients. We will specifically direct our attention to 15 genes associated with antibiotic resistance, assigned to the mutational resistome of *P. aeruginosa* [4, 5], or associated with ‘antibiotic resistance and susceptibility’ according to the *Pseudomonas aeruginosa* Community Annotation Project (PseudoCAP) [6–8].

## *P. aeruginosa* bacterium

*P. aeruginosa* is a Gram-negative bacterium, which is isolated from a variety of environments such as soil, water, plants and animals, as part of the natural bacterial microbiota, but also is known as an opportunistic pathogen. This dual life-style is likely attributable to its large genome (~6–7 Mb) [9], which also includes a range of different mechanisms associated with antibiotic resistance [10]. Moreover, as a consequence of its inherent antibiotic resistance and its pathogenic potential, it has been described as a ‘priority pathogen’ by the World Health Organization [11].

## Studies of antibiotic resistance

Antibiotic resistance has been, and still is, extensively investigated in laboratory experiments [10, 12–15]. These experiments generally provide a simple causality between genetic change(s) and resistance phenotype, as there is usually only one or few variable selective pressures (e.g. antibiotics) present, which is optimal for studying primary mechanisms of antibiotic resistance and identifying the associated genetic determinants.

However, the environments in which the antibiotics are meant to be used, and in which resistance interferes with treatment, are never as simple as in a test tube (Fig. 1). The human or animal host represents arrays of selective forces, which the invading bacteria meet and have to adapt to, in order to establish an infection. Some of these host-associated challenges resemble the selective pressure from antimicrobials, e.g. presence of toxins and antimicrobial compounds expressed by the commensal flora or by the immune system [16, 17] (Fig. 1). Therefore, to understand antibiotic resistance as it develops in the host, both through primary and secondary selection, we need to contextualize the findings from the laboratory studies with studies of complex natural systems. One excellent infection model system to carry out such investigations is persistent airway infection in CF patients.

## Cystic fibrosis

CF is an autosomal recessive inherited disease caused by a malfunctioning chloride channel resulting in an imbalance in salt concentrations across the epithelial layers, which is particularly problematic on mucosal surfaces, where the mucus layer gets thick and sticky due to dehydration. In the lungs of CF patients, this results in reduced clearance of airway secretions and, thus, CF patients are prone to airway infections. One of the most frequent infectious agents is *P. aeruginosa* [18].

CF patients routinely attend out-patient clinics, where lung samples are cultured to identify and monitor airway infections, and from which bacterial isolates are collected. Such isolates stored over time provide longitudinal bacterial libraries, useful for investigations of the adaptive trajectories of persistent infections [1–3, 5]. There are several such examples in the literature, which document how historical contingencies and epistatic interactions [1, 19], cooperation and cheating in bacterial populations [20], horizontal gene transfer and gene loss in natural populations [21], and antibiotic resistance development [4, 22] can be documented from investigations of genomic and phenotypic changes over time. Longitudinal bacterial isolates provide opportunities to study the evolution of antibiotic resistance in complex and dynamic environments with clinically relevant antibiotic concentrations. Such investigations provide relevant contexts for comparisons with more controlled laboratory experiments characterized by a single or a few specific selective pressures.

## Initial Adaptation to the Human Host

Despite early treatment with antibiotics, initial colonization of CF airways does not seem to result in resistance development in *P. aeruginosa* populations [22, 23]. Instead, establishment of an infection probably depends much more on adaptation to a range of other selective factors within the human host such as: the immune system, altered nutrient availability, fluctuating oxygen concentrations and the composition of the indigenous microbiota [16, 24–28] (Fig. 1).

If antibiotic resistance plays a minor role in the initial colonization of the CF human host, it is in fact surprising that 15 of the 52 pathoadaptive genes identified as particularly frequent targets for mutations in early CF airway infections [1] are associated with antibiotic resistance [4, 5]. However, all of these 15 mutated genes are additionally associated with other important infection-associated phenotypes (PseudoCAP) (Table 1) and, thus, could be selected for by conditions in the host other than the presence of antibiotics.

**Table 1.** Fifteen genes associated with antibiotic resistance These genes [4, 5] were selected because they have also been found to be important for the adaptation of *P. aeruginosa* to the airways of CF patients [1]. The PseudoCAP functions associated with each gene are marked by coloured boxes representing the different functions as noted in the key.

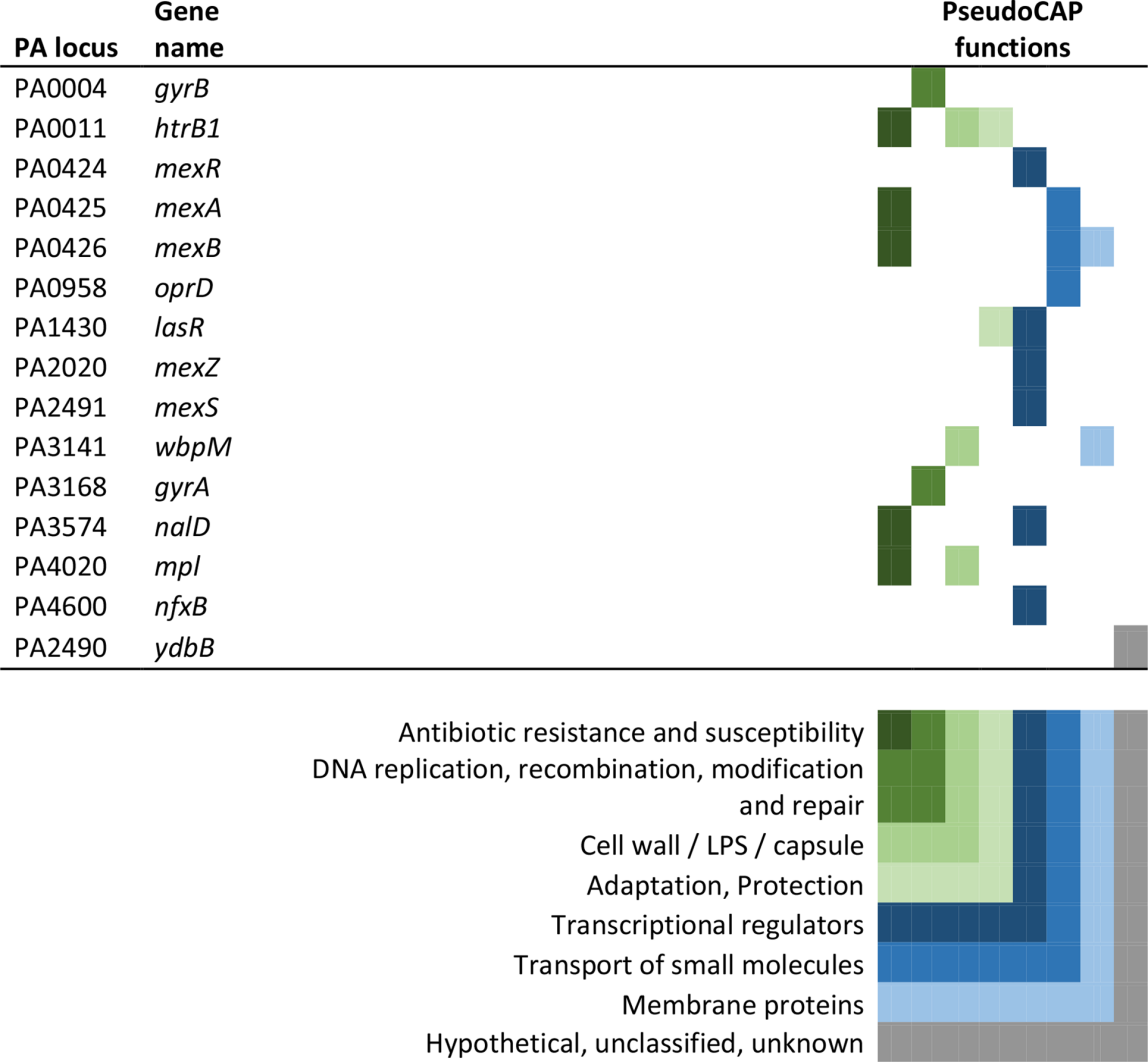

For 12 of the resistome-associated pathoadaptive genes, the number of phenotypic traits not directly linked to antibiotic resistance is striking, and highlight the diverse selective pressures that the bacteria face in the initial stages of infection. Specifically, we find genes associated with cell-wall biosynthesis (*htrB1* [15], *wbpM* [29], *mpl* [30]), efflux pumps and porins (*mexR, mexA, mexB* [14], *mexZ* [31], *mexS* [32, 33], *nalD* [13], *oprD* [12]) and quorum sensing (*lasR* [34]). In all cases, mutations in these genes may change antibiotic susceptibility, but they are also linked to responses towards other environmental stressors.

When entering the human host, the bacteria will encounter the innate immune system and the indigenous microbiota. The innate immune system will likely be activated by cell-wall components of the bacteria, such as the lipopolysaccharides (LPS), and mutations in genes related to the LPS will help the bacteria to ‘hide’ from the immune system. Additionally, the peptidoglycan of the cell wall and efflux pumps will provide the infecting bacteria with protection against toxic compounds produced by other micro-organisms or other environmental compounds (e.g. heavy metals, quorum sensing signals, etc.). These effects have been reviewed elsewhere [24, 35, 17]. Interestingly, mutations in the negative regulator of the MexXY efflux pump (*mexZ*) outnumber all other pathoadaptive mutations [1] in this collection, and the gene has also been found to be highly important in other studies, as reviewed previously [36]. It has been associated with resistance to aminoglycosides and fluoroquinolones [37, 38], but in most cases *mexZ* mutations have been found to be associated with resistance levels far below the EUCAST breakpoints, pointing to yet another clinical challenge when comparing genotypes and phenotypes [39].

Of the 15 resistome-associated pathoadaptive genes, 3 are likely to accumulate mutations as a direct consequence of antibiotic selection. For example, mutations in the genes *gyrA*, *gyrB* and *nfxB* have been linked specifically to resistance against a distinct group of antibiotics: the quinolones, which include ciprofloxacin [40–42]. Accordingly, mutations in these genes have been identified in isolates with increasing ciprofloxacin resistance over time [22]. However, in spite of a direct connection between gyrase and the antibacterial mode of action of ciprofloxacin, mutations in either of the gyrase-encoding genes do not always result in the development of resistance. This is even observed when bacteria are under high levels of selection for resistance development (i.e. high levels of antibiotics in the environment). As shown by Bartell *et al*. [22], 78 % of such mutated isolates showed ciprofloxacin resistance (based on the EUCAST breakpoint), leaving 22 % of the mutations without any antibiotic-resistance phenotype. Others have found isolates with the same *gyrA* mutation to result in MIC values varying more than 1000-fold [43], highlighting the importance of the genetic background in which the mutations are found.

## Concluding remarks

We have described parts of the wide repertoire that can be deployed by *P. aeruginosa* to achieve a high level of infection success and persistence (fitness) in the host. Many of these mechanisms are associated with antibiotic resistance but, as we have discussed here, it is far from clear that there is a direct causality between specific mutations and specific phenotypes. Therefore, we conclude that despite the usefulness of full-genome sequences of pathogenic bacteria as part of the diagnostic repertoire in the clinic, we still need to take into consideration the following: (i) that mutations are fixed in bacterial populations as consequences of the specific environments in which the bacteria are growing (selection forces), and (ii) that the specific mutations cause phenotypic changes that depend on the rest of the genomic configuration (epistatic impacts). It is consequently doubtful that genomic diagnostics can fully substitute for phenotypic characterization, and that depending entirely on genomics in the clinic may result in erroneous diagnoses and resulting therapeutic extrapolations.
